# Gold nanoparticle thin films fabricated by electrophoretic deposition method for highly sensitive SERS application

**DOI:** 10.1186/1556-276X-7-613

**Published:** 2012-11-06

**Authors:** Sheng-Qing Zhu, Tong Zhang, Xin-Li Guo, Qi-Long Wang, Xuefeng Liu, Xiao-Yang Zhang

**Affiliations:** 1 School of Electronic Science and Engineering, Southeast University, Nanjing, 210096, People's Republic of China; 2 Key Laboratory of Micro-Inertial Instrument and Advanced Navigation Technology, Ministry of Education, Nanjing, 210096, People's Republic of China; 3 Suzhou Key Laboratory of Metal Nano-Optoelectronic Technology, Suzhou Research Institute of Southeast University, Suzhou, 215123, People's Republic of China; 4 School of Materials Science and Engineering, Southeast University, Nanjing, 211189, People's Republic of China; 5 Institute of Optics and Electronics, CAS, PO Box 350, Chengdu, Shuangliu, 610209, China

**Keywords:** Gold nanoparticle, Electrophoretic deposition, SERS

## Abstract

We report an electrophoretic deposition method for the fabrication of gold nanoparticle (GNP) thin films as sensitive surface-enhanced Raman scattering (SERS) substrates. In this method, GNP sol, synthesized by a seed-mediated growth approach, and indium tin oxide (ITO) glass substrates were utilized as an electrophoretic solution and electrodes, respectively. From the scanning electron microscopy analysis, we found that the density of GNPs deposited on ITO glass substrates increases with prolonged electrophoresis time. The films possess high mechanical adhesion strength and exhibit strong localized surface plasmon resonance (LSPR) effect by showing high SERS sensitivity to detect 1 × 10^−7^ M rhodamine 6 G in methanol solution. Finally, the relationship between Raman signal amplification capability and GNP deposition density has been further investigated. The results of our experiment indicate that the high-density GNP film shows relatively higher signal amplification capability due to the strong LSPR effect in narrow gap regions between the neighboring particles on the film.

## Background

Films composed of noble metal nanoparticles (typically, Au or Ag) currently have attained wide popularity and aroused intense research interest in nanotechnology due to the intriguing optical properties introduced by localized surface plasmon resonances (LSPRs). LSPRs, which are optical phenomenona arising from the collective oscillation of conduction electrons in noble metal nanoparticles when the electrons are disturbed from their equilibrium positions, lead to enormous optical local-field enhancement at the nanoscale and obtain potential applications in many fields such as chemical or biosensors [1-4], solar cell designs [5-8], and surface-enhanced Raman scattering (SERS) [9-11].

Due to the exceptional optical properties of noble metal nanoparticle films, many available methods for the fabrication of these films have been proposed in the past two decades. In order to obtain a strong LSPR effect, it is often necessary to engineer the particle deposition density on substrates for practical application because the deposition density can greatly affect the optical property of the films [12]. The most recently available methods for the fabrication of these films include electron beam lithography and nanoimprint lithography, both can completely control the micromorphology of the nanostructures for the design with unique LSPR spectrum [13,14]. However, these methods require sophisticated fabrication equipment and are limited by either expensive cost or small sample size in practical applications. Instead, some simpler bottom-up approaches based on self-assembly, e.g., Langmuir-Blodgett, dip coating, and electrochemical deposition, have shown great conveniency in large-scale fabrication and much less defectivity [15-19]. Such techniques can surely produce noble metal nanoparticle thin films with large areas, but the resulting films usually lack mechanical adhesion strength of nanoparticles to substrate materials required for device construction.

In this paper, we report a rapid, simple, and room-temperature electrophoretic deposition method using gold nanoparticle (GNP) sol as an electrophoretic solution to fabricate sensitive GNP films with high mechanical adhesion strength. It is found that the GNP deposition density on the films can be adjusted by changing the electrophoresis time, and the sub-10-nm gaps can be formed between the neighboring particles on the film for the relatively longer electrophoresis time. Finally, we demonstrate the excellent signal enhancement ability of these GNP thin films as substrates in SERS measurements and find that rhodamine 6 G (R6G) molecules can be detected at very low concentrations using these films.

## Methods

**Figure 1:**
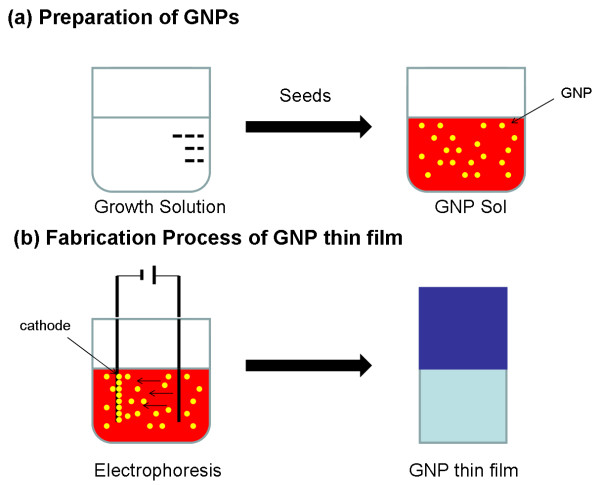
Schematic illustration of (a) preparation of GNP sol and (b) fabrication process of GNP thin film.

Figure 1 shows the scheme of the fabrication process of the GNP thin films. The process contains two steps: In the first step, the GNP sol, as an electrophoretic solution for GNP thin films, was synthesized by a seed-mediated method [20,21]. As shown in Figure 1a, the GNP sol was synthesized by adding the seed solution (aqueous solution of cetyltrimethylammonium bromide (CTAB), NaBH_4_, and HAuCl_4_) into the growth solution (aqueous solution of CTAB, ascorbic acid, and HAuCl4). The resulting solution was gradually transformed into red color. In this synthesis procedure, bilayers of CTAB, which was used as surfactant, were formed on the GNPs' surface, and these GNPs kept a net positive charge to prevent random aggregation between particles. After growing over one night, the GNP sol was centrifuged at 14,000 rpm for 10 min and then redispersed into deionized water (>18.0 MΩ cm) to remove redundant reactants in the solution. In the second step, electrophoretic deposition method was employed for film preparation. As illustrated in Figure 1b, two indium tin oxide (ITO) glass substrates (30 × 10 × 0.6 mm) were immersed into the GNP sol (approximately 10^15^ particles L^−1^) and used as cathode and anode, with an interelectrode distance of 3 mm, for electrophoresis. During the experiment, electrophoresis was carried out by applying a voltage of 4.5 V at room temperature. Under otherwise equal conditions, three film samples, S1, S2, and S3, were prepared with different electrophoresis times, i.e., 8, 2, and 1 min, respectively. When the electrophoresis process was finished, the samples were rinsed with deionized water (>18.0 MΩ cm) and then baked on a hot plate at 60°C for 30 min to remove the residual solution.

## Results and discussion

**Figure 2:**
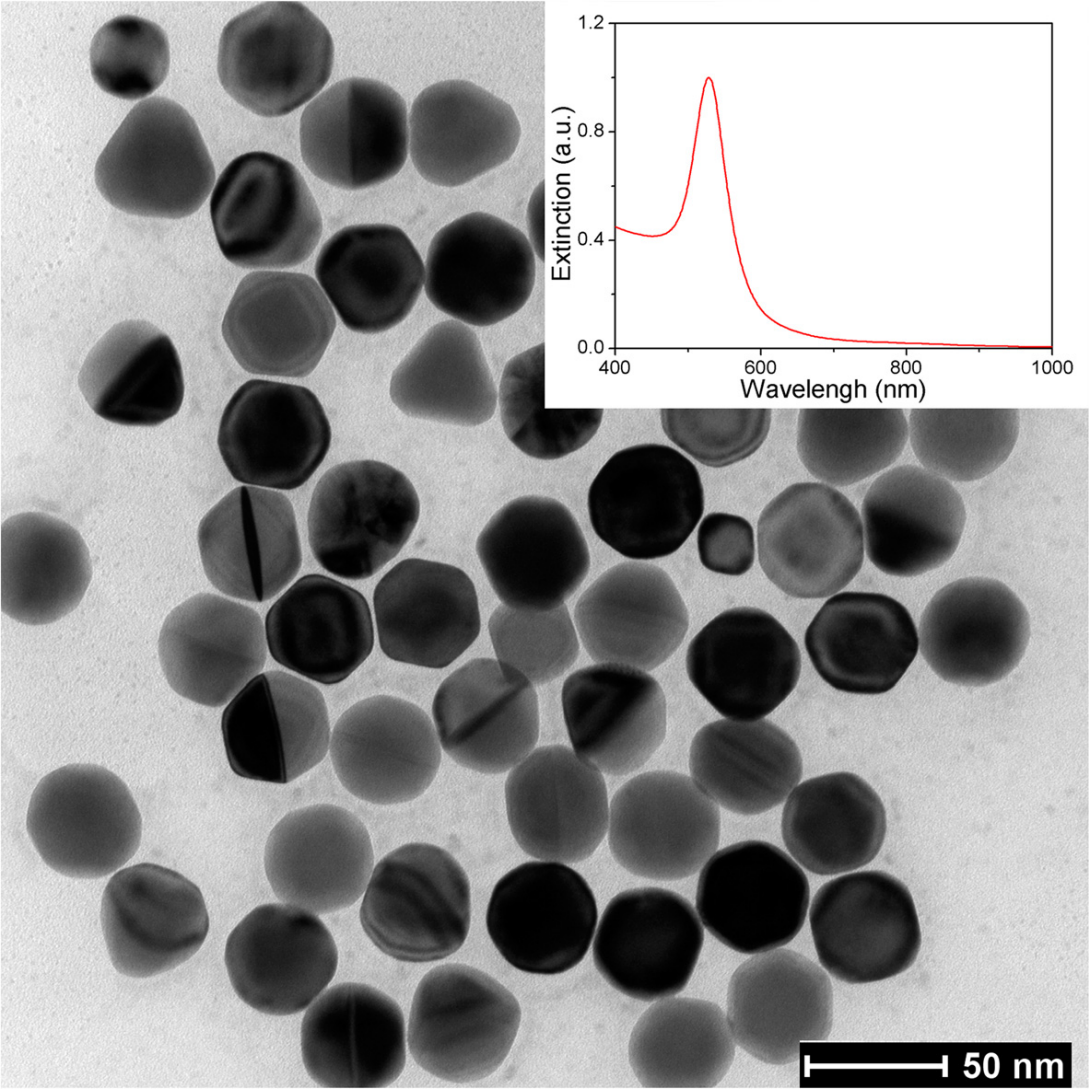
**TEM image of GNPs synthesized by seed-mediated method.** The inset gives the measured extinction spectrum of the GNP sol.

Firstly, we characterized the micromorphology of the GNPs in the electrophoresis solution using a transmission electron microscope (TEM). Figure 2 shows the TEM image of GNPs in the sol. It is found that the GNPs are single crystals with a nearly uniform spherical shape and well dispersed in the solution due to the Coulomb repulsion effect, which was introduced by the bilayers of CTAB on GNPs' surfaces [22]. The average size of GNP is between 43 and 47 nm, which is dependent on the amount of gold seeds added during the synthesis process. Then, we also examined the plasmon resonance characteristic of GNP sol using a UV–vis-NIR spectrophotometer. The extinction spectrum of the GNP sol (the inset in Figure 2) indicates that the sol has a single narrow plasmon resonance peak at 531 nm, and this result indirectly proved the excellent control of micromorphology of the GNPs in the synthesis process.

**Figure 3:**
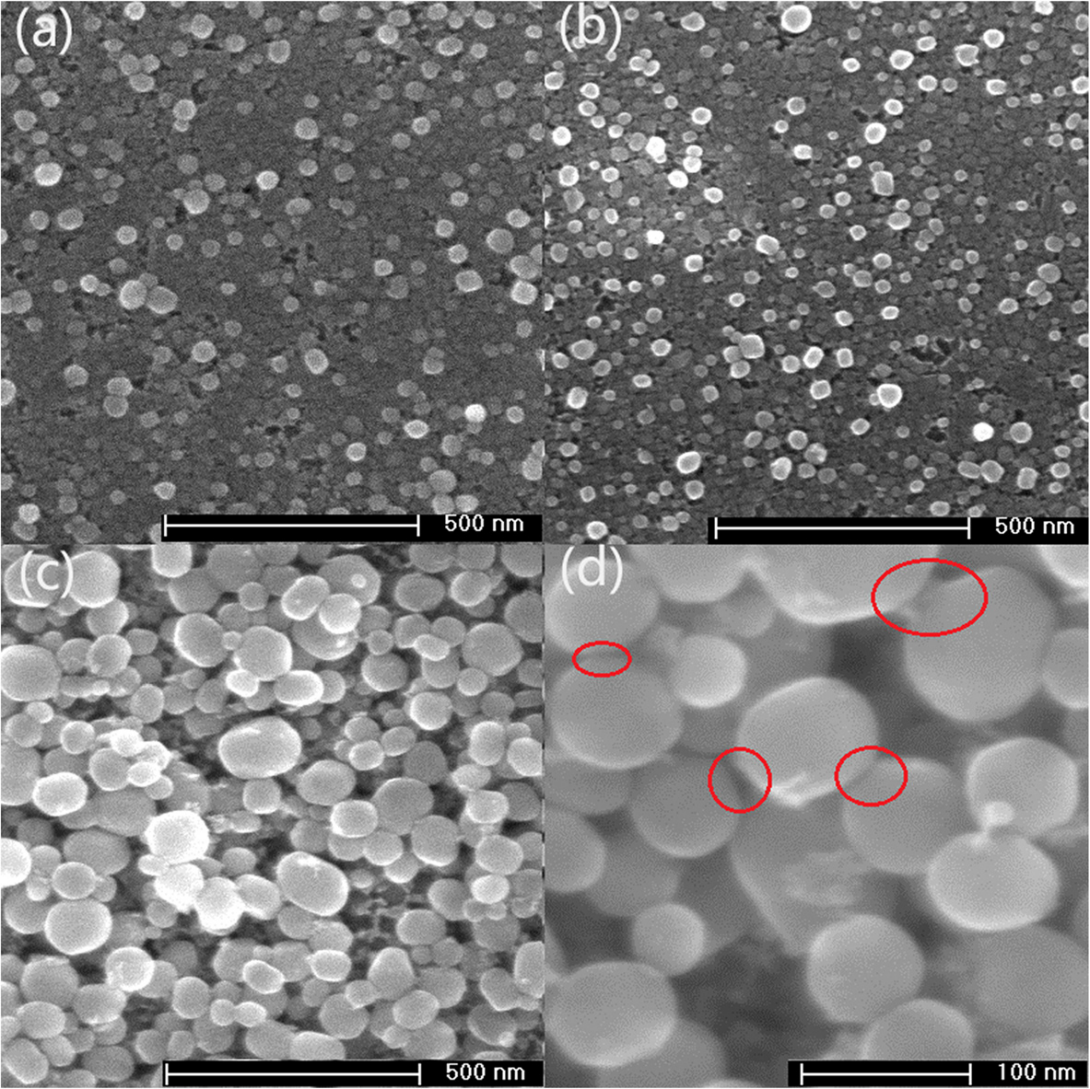
**Comparison of top-view FESEM images of the three samples: (a) S3, (b) S2, (c) S1.** (**d**) High-resolution FESEM image of sample S1. The positions (red circles) indicate that sub-10-nm gaps have been formed between the neighboring particles on this film.

In this experiment, a simple principle was utilized to form GNP films: because the CTAB-wrapped nanoparticles in the solution possess a net positive charge, under an external electric field condition, the particles moved to the cathode and attached onto the ITO glass. To investigate the micromorphology of GNP thin films deposited on the ITO glass substrates, field emission scanning electron microscopy (FESEM) was performed. Figure 3 shows AuNP depositions from dispersed distribution to multilayered close packing which are achieved by varying the electrophoresis time. For a short electrophoresis time (1 or 2 min), the particles on samples S2 and S3 were well uniformly separated over the surface of the ITO glass (Figure 3a,b). When the electrophoresis time was prolonged to 8 min (Figure 3c,d), the GNP thin film on sample S1 was gradually transformed into a multilayered close-packed structure in the microscale. Despite the same electrophoresis solution in the fabrication process for the three films, the GNPs deposited on the three samples have different sizes with different electrophoresis times. Most particles on samples S2 and S3 fall in the size range of 45 to 50 nm, which is close to the particle size in the original deposition solution. For sample S1, however, the typical size of GNP on the film is 61 nm, which is both larger than that of the original particles in the GNP sol and the ones on samples S2 and S3. This particle size change may be attributed to the recrystallization of the Au^+^ ions that resided in the GNP sol. We suspect that in the electrophoresis process, an electrochemical deposition process also occurred simultaneously and the particle size gradually increased because some Au^+^ ions were reduced into Au atoms and attached on the original GNPs on the cathode surface. From the high-resolution FESEM image (Figure 3d), we found that the particles nearly retain the shape of the original ones in the deposition solution (see Figure 2). Therefore, this shape maintenance property gives us a message that the shape and size of GNPs on films can be changed by altering the original GNPs, e.g., nanorod, nanotriangular, and nanocubic, which can be easily synthesized by adjusting the concentrations of reactants in a seed-mediated method [20,22]. From the high-resolution image, it is also found that the sub-10-nm gaps (the red circles in Figure 3d), which play an important role in SERS applications [17,23], were formed between the neighboring particles on sample S1.

The mechanical adhesion strength of the GNP thin films was tested by ultrasonication in deionized water (>18.0 MΩ cm). For comparison, an extra sample was prepared by directly depositing a droplet of the GNP sol onto an ITO glass substrate, and GNPs remained on the surface of the substrate after evaporating the sol under ambient conditions. FESEM observations (not shown in figure) were carried out at the same positions on the substrates both before and after applying 5-min ultrasonication on the GNP thin films and the reference one. We found that the GNPs on thin films fabricated by electrophoretic deposition method were resistant to the ultrasonication process. The micromorphology had little change on the surface of the thin film after applying ultrasonication. In contrast, GNPs on the reference sample were not able to resist the ultrasonication process, and a retention rate below 35% was observed. This contrasting result indicates that the GNP thin films fabricated by electrophoretic deposition method possess superior mechanical adhesion strength.

Raman spectroscopy is not only a powerful analytical technique in composition analysis, but also a testing tool to examine the LSPR effect of GNP thin films. The Raman spectra have been intensively used in the field of identification of organic molecules from their vibration spectra at very low concentrations. In the past two decades, many studies have revealed that noble metal nanoparticle films with a strong LSPR effect can amplify the Raman signals in biological detection process [9-11]. In order to evaluate the Raman-enhancing capability of the GNP thin films fabricated by electrophoretic deposition method, various R6G solutions with different concentrations were prepared because they have been extensively studied in prior literatures [1,24]. R6G is a strongly fluorescent xanthene derivative that is a yellowish heterocyclic compound and shows a molecular resonance Raman effect when excited into its visible absorption band. In this experiment, a confocal Raman microscope/spectrometer with a × 50 objective was used for the Raman measurements. An Ar laser with a wavelength of 514 nm was employed for the excitations. The laser power focused on the samples was measured to be 1.5 mW/cm^2^. The laser beam size focused on the samples was 2 μm in diameter, and the acquisition time was 10 s. For comparison, R6G in methanol solutions with different concentrations were dropped onto GNP thin films and bare ITO glass substrates with no nanoparticles. Raman spectra were recorded after drying the solvent. For bare ITO glass substrates, the resulting spectra show no discernable Raman peaks of R6G molecules at the concentration of 10^−4^ M (not shown in figure). Until the concentration increased to 10^−3^ M, a few weak peaks can be observed. Therefore, when the R6G concentration is equal to or less than 10^−4^ M, peaks associated to R6G molecules cannot be detected in the Raman measurement.

**Figure 4:**
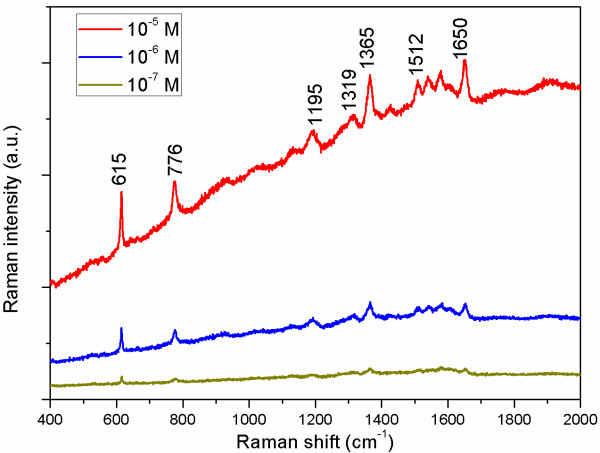
SERS spectra of the different concentrations of R6G adsorbed on the surface of sample S1.

In order to examine the enhancement capability of GNP thin films to Raman signal, three R6G solutions with different concentrations of 10^−5^, 10^−6^, and 10^−7^ M (which cannot be detected on bare ITO glass substrates with no nanoparticles) have been applied at three different points on sample S1, the film with the highest GNP density. As shown in Figure 4, salient surface-enhanced characteristic peaks of R6G molecules on this film can be seen noticeably, even if the concentration drops dramatically to 10^−7^ M. The band at 615 cm^−1^ is due to plane bending of the C-C-C ring, whereas the band at 776 cm^−1^ has been assigned to out-of-plane bending of the hydrogen atoms of the xanthene skeleton [25]. The peak at 1,195 cm^−1^ is associated with C-C stretching vibrations, and those at 1,319, 1,365, 1,512, and 1,650 cm^−1^ correspond to aromatic stretching vibrations [26]. Thus, these results indicate that the R6G solution at the very low concentration of 10^−7^ M can be successfully identified using sample S1 as a sensitive SERS substrate.

In previous works [9,11], a plasmon resonance theory is widely used to explain SERS enhancement. In this theory, the LSPR effect of metal nanoparticles plays an important role in Raman signal amplification because the electromagnetic field strength on the metal particle surface becomes strongest when LSPR is excited. Metal films that serve as SERS substrates often need high particle deposition density to form ‘hot spots’ in the narrow gap regions between neighboring particles, which can harvest high electromagnetic field to enhance the Raman signal [15]. From Figure 3d, one can find that many narrow gaps with sub-10-nm distance are clearly formed between neighboring particles on the film. These regions can produce a strong LSPR effect on the surface of the thin film when GNPs are excited by the incident light and enable the Raman characteristic peaks of R6G at a very low concentration to be easily detected. Compared with the previous works [17,27], Raman signal enhancement is also observed in similar GNP films because of the presence of narrow gaps. For instance, in [17], R6G at an extremely low concentration of 10^−9^ M was successfully identified using nanosphere arrays with sub-10-nm gaps as sensitive SERS substrates.

**Figure 5:**
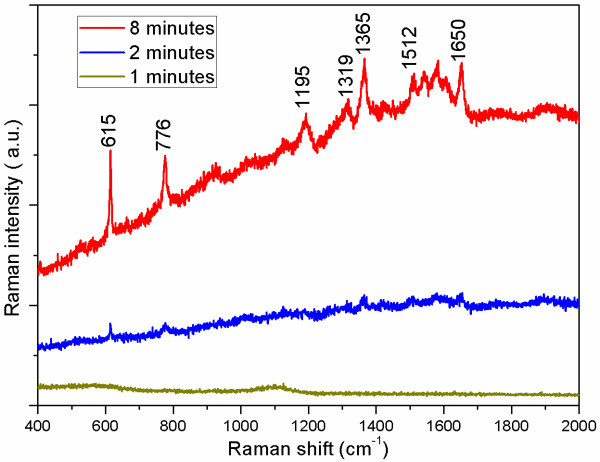
**SERS spectra of R6G (10**^**−5**^ **M) obtained from the surface of the three samples.**

We have showed remarkable SERS signal amplification effect using sample S1 with the highest nanoparticle deposition density. To confirm the influence of the GNP deposition density on Raman signal measurement, we have carried out SERS studies on the other two films, S2 and S3. For simplicity, an R6G solution with a concentration of 10^−5^ M was applied on the two films. From the resulting Raman spectra (Figure 5), one can see that although sample S2 still can generate the characteristic peaks of R6G, it is much weaker than sample S1. For sample S3, there is even no discernable Raman peak of R6G shown on the Raman spectrum. This behavior of losing Raman signal amplification capability implies that the LSPR effects on samples S2 and S3 are much weaker than that of sample S1. Obviously, these results show an excellent agreement of the micromorphology of the three samples because the densities of GNPs deposited on samples S2 and S3 were much lower than that of sample S1. Thus, the narrow gaps located between neighboring particles on samples S2 and S3 were relatively fewer and caused the weaker Raman signals.

## Conclusions

In summary, a rapid, simple, and room-temperature electrophoretic deposition method has been proposed to fabricate GNP thin films with high mechanical adhesion strength. Sub-10-nm gaps between neighboring particles have been formed when GNP deposition density increases. The films made by this method exhibit a high Raman signal due to the strong LSPR effect, which is produced in the narrow gap regions. This design of GNP thin films with a highly sensitive SERS-active property may provide a new framework for the fabrication of large-area SERS-based sensors.

## Competing interests

The authors declare that they have no competing interests.

## Authors' contributions

S-QZ carried out the design and the characterization of GNP thin films, performed the SERS analysis, and drafted the manuscript. TZ, X-LG, Q-LW, XL, and X-YZ read and contributed in the improvement of the manuscript. All authors read and approved the final manuscript.
